# Examining the Role of Pre-Operative Retrolisthesis in Single-Level Lumbar Fusion: Impact on Reoperation Rates

**DOI:** 10.3390/jcm15114063

**Published:** 2026-05-24

**Authors:** Hershil Alkesh Patel, Rohan I. Suresh, Sapan Patel, Abel K. Lindley, Ethan Yang, Gerald Kidd, Evan Honig, Ryan Curto, Usman Zareef, Hans Prakash, Amil Sahai, Husni Alasadi, Alexander Padovano, Louis J. Bivona, Daniel Cavanaugh, Eugene Y. Koh, Steven C. Ludwig, Julio J. Jauregui

**Affiliations:** Department of Orthopaedics, Division of Spine Surgery, University of Maryland School of Medicine, Baltimore, MD 21201, USA; hershilpatel@som.umaryland.edu (H.A.P.); rohansuresh10@gmail.com (R.I.S.); sapan.patel@som.umaryland.edu (S.P.); abel.lindley@som.umaryland.edu (A.K.L.); ethan.yang@som.umaryland.edu (E.Y.); gkidd@som.umaryland.edu (G.K.); ehonig@som.umaryland.edu (E.H.); ryan.curto@som.umaryland.edu (R.C.); uzareef@som.umaryland.edu (U.Z.); hprakash@som.umaryland.edu (H.P.); amil.sahai@som.umaryland.edu (A.S.); alexander.padovano@som.umaryland.edu (A.P.); lbivona@som.umaryland.edu (L.J.B.); dcavanaugh@som.umaryland.edu (D.C.); ekoh@som.umaryland.edu (E.Y.K.); sludwig@som.umaryland.edu (S.C.L.)

**Keywords:** lumbar fusion, retrolisthesis, adjacent segment disease, revision surgery, spinopelvic alignment

## Abstract

**Background/Objectives:** Lumbar fusion for degenerative pathology is increasingly common, yet revision surgery, often due to adjacent segment disease, remains a major concern. While sagittal alignment has been widely studied, the role of preoperative lumbar retrolisthesis is less clear. This study evaluated the prevalence, distribution, and clinical significance of preoperative retrolisthesis in patients undergoing single-level L4–L5 fusion, with a focus on its association with revision surgery. **Materials and Methods:** A retrospective cohort study of 116 adult patients undergoing single-level L4–L5 posterior fusion with multilevel decompression from 2018 to 2022 was performed. Retrolisthesis was defined as posterior translation ≥ 2 mm and assessed across lumbar levels. Patients were stratified by revision status. Statistical comparisons and Cox proportional hazards models were used to evaluate associations between retrolisthesis burden, radiographic parameters, and revision risk. **Results:** Preoperative retrolisthesis was present in 78.4% of patients, most commonly at L3–L4 and L2–L3, and frequently involved multiple levels. Presence of retrolisthesis alone was not associated with revision (85.7% vs. 77.5%, *p* = 0.73). However, patients with ≥3 retrolisthesis levels demonstrated reduced revision-free survival, with a higher hazard of revision on univariate analysis (HR 3.65, *p* = 0.055). In multivariate analysis, ≥3 levels (HR 5.50, *p* = 0.018) and greater absolute L4–S1 lordosis change (HR 1.09 per degree, *p* = 0.031) were independent predictors of revision. **Conclusions:** Retrolisthesis is highly prevalent in patients undergoing L4–L5 fusion but is not predictive of revision when considered alone. A greater multilevel retrolisthesis burden, particularly when combined with larger segmental lordosis change, may represent a mechanically vulnerable spine phenotype associated with increased revision risk. These findings should be interpreted as exploratory and require validation in larger cohorts.

## 1. Introduction

Lumbar fusion is commonly performed in the United States for degenerative lumbar spine disease, with national database studies demonstrating a marked increase in elective lumbar fusion volume over the past two decades [1,2,3]. Degenerative lumbar spinal stenosis is a major source of pain, disability, and impaired function in older adults and remains the most common indication for spine surgery in patients older than 65 years [4]. Within this spectrum of pathology, degenerative spondylolisthesis is a particularly common indication for operative treatment and most frequently occurs at L4–L5, a level subject to substantial segmental motion and mechanical loading. Anterolisthesis refers to anterior translation of a vertebral body relative to the subjacent vertebra, whereas retrolisthesis refers to posterior translation relative to the subjacent vertebra [5,6]. Accordingly, L4–L5 fusion is commonly performed in patients with symptomatic stenosis and degenerative spondylolisthesis when neural compression, mechanical back pain, and radiographic instability warrant operative decompression and stabilization [7,8,9].

Despite advances in surgical technique and perioperative care, revision after lumbar fusion remains an important clinical problem, with adjacent segment disease (ASD) representing one of the most recognized late modes of failure [10,11]. The development of ASD is widely considered multifactorial, reflecting both the natural history of lumbar degeneration and fusion-related changes in adjacent-level biomechanics, including increased motion, intradiscal pressure, and facet loading across unfused segments [10,11]. Accordingly, prior work has focused heavily on alignment-based risk factors, with multiple studies demonstrating that sagittal malalignment, particularly pelvic incidence-lumbar lordosis (PI-LL) mismatch, is associated with symptomatic adjacent-level disease and revision after lumbar fusion [12,13,14]. In contrast, comparatively less attention has been paid to preoperative retrolisthesis. In the lumbar spine, retrolisthesis has been described as a potential marker of disc degeneration, low pelvic incidence, and sagittal compensatory mechanisms, and has been inconsistently associated with pain and dysfunction in non-fusion populations [15,16,17,18]. However, its prevalence and clinical relevance in patients undergoing single-level lumbar fusion, particularly as a marker of multilevel biomechanical vulnerability predisposing to later revision, remain poorly defined.

Accordingly, the purpose of this study was to evaluate the prevalence, distribution, and clinical significance of preoperative lumbar retrolisthesis in patients undergoing single-level L4–L5 fusion for degenerative pathology. Specifically, we sought to determine: (1) the prevalence of retrolisthesis at any lumbar level in this population; (2) whether preoperative retrolisthesis, including adjacent-level retrolisthesis, is associated with an increased risk of revision surgery; and (3) whether patients with retrolisthesis demonstrate distinct radiographic and clinical characteristics compared with those without retrolisthesis. We hypothesized that retrolisthesis would be common in this population and that greater retrolisthesis burden would be associated with a higher likelihood of revision, reflecting underlying multilevel biomechanical vulnerability.

## 2. Materials and Methods

### 2.1. Study Design

We conducted a retrospective cohort study to characterize the prevalence and distribution of adjacent-level retrolisthesis in patients undergoing single-level L4–L5 posterior spinal fusion with multilevel decompressive laminectomy for degenerative lumbar pathology, and to evaluate whether retrolisthesis (overall or at levels adjacent to the fusion construct) was associated with subsequent revision surgery. All procedures were performed at the University of Maryland Medical Center between January 2018 and December 2022. The institutional review board approved this study, and the requirement for informed consent was waived, given the retrospective design.

### 2.2. Patient Population and Selection Criteria

We identified consecutive adult patients (age 18 years or older) who underwent single-level L4–L5 posterior spinal fusion with concurrent multilevel decompressive laminectomy during the study period. Multilevel laminectomy was defined as decompression involving two or more vertebral laminae, including the L4 and L5 laminae at minimum. Inclusion required a primary diagnosis of degenerative L4–L5 spondylolisthesis with associated stenotic symptoms and at least one postoperative clinical follow-up visit. We excluded patients with non-degenerative indications such as trauma, infection, tumor, or primary deformity correction, as well as patients undergoing multilevel fusion or those lacking adequate preoperative standing lateral radiographs. After excluding 1 patient whose revision was for a non-degenerative wound complication, 116 patients constituted the final analytic cohort. Revision surgery was defined as any return to the operating room at the index surgical level or adjacent levels for symptomatic adjacent segment disease or adjacent stenosis after the index operation. All revision events included in the final analysis were performed for symptomatic adjacent segment disease/adjacent stenosis in patients presenting with radicular pain and radiographic pathology warranting operative management. Revisions for non-degenerative causes, including wound infection, isolated hardware failure, screw loosening, or rod breakage, were not included as revision endpoints. Subsequent radiographic spondylolisthesis was not analyzed as a separate endpoint; when present, adjacent-level degenerative instability was considered part of the broader adjacent segment disease process.

### 2.3. Operative Technique

All patients underwent single-level L4–L5 posterior fusion with concurrent open multilevel decompressive laminectomy through a standard posterior midline approach. Decompression was performed as a traditional open laminectomy involving two or more vertebral laminae, including L4 and L5 at minimum, with extension to additional levels based on the distribution of stenosis. Interlaminar, unilateral laminotomy for bilateral decompression, and over-the-top decompression techniques were not used. Posterior fusion was performed at L4–L5 according to the treating surgeon’s standard technique.

### 2.4. Data Collection

We extracted demographic, clinical, operative, and radiographic data from the electronic medical record and operative reports. Demographic variables included age at surgery, sex, race, body mass index (BMI), and the Charlson Comorbidity Index (CCI). Age at surgery was available for 83 of 116 patients (71.6%). Comorbidity burden was characterized by individual comorbidities (diabetes mellitus, hypertension, osteoporosis, chronic kidney disease, cancer history, and chronic steroid use) and American Society of Anesthesiologists (ASA) physical status classification. Smoking status was recorded as current, former, or never. Preoperative symptoms assessed included back pain, leg pain, bilateral leg symptoms, and motor weakness on neurologic examination. Operative variables included surgery length, estimated blood loss (EBL), intraoperative complications, hospital length of stay (LOS), and hospital complications. Revision surgery was defined as any return to the operating room at the index surgical level or adjacent levels for a new procedure performed after the index operation.

### 2.5. Radiographic Measurements

Standing lateral radiographs were reviewed at two time points: preoperative and immediate postoperative (within 72 h of surgery). Preoperative measurements were available for all 116 patients. Immediate postoperative measurements of sacral slope (SS), L1–S1 lumbar lordosis (LL), and L4–S1 segmental lordosis were available for 75 patients (64.7%), with data for all 14 revision patients but only 61 of 102 non-revision patients (59.8%). We measured pelvic incidence (PI), SS, L1–S1 LL, and L4–S1 lordosis. PI-LL mismatch was calculated as the arithmetic difference between PI and L1-S1 lordosis. Because PI is an anatomical constant that does not change with surgical intervention, postoperative PI was set equal to preoperative PI. Change scores (delta values) were computed as the difference between immediate postoperative and preoperative measurements for SS, lordosis, and PI-LL mismatch. All radiographic measurements were performed by trained reviewers using calibrated digital imaging software.

### 2.6. Retrolisthesis Classification

Retrolisthesis was assessed at each lumbar motion segment from L1–L2 to L5–S1 on preoperative standing lateral radiographs. Retrolisthesis was assessed at each lumbar motion segment from L1–L2 to L5–S1 on preoperative standing lateral radiographs. For each motion segment, anterolisthesis was defined as anterior translation of the superior vertebral body relative to the inferior vertebral body, whereas retrolisthesis was defined as posterior translation. A segment was classified as retrolisthetic if posterior translation measured at least 2 mm, consistent with the conventional radiographic threshold. We recorded the total number of retrolisthetic levels per patient and the specific levels involved. Adjacent retrolisthesis was defined as retrolisthesis at L3–L4 (immediately cephalad to the fusion construct) or L5–S1 (immediately caudal). Remote retrolisthesis was defined as retrolisthesis at L1–L2 or L2–L3. All retrolisthesis identified in this cohort were low-grade/Grade I. No patient demonstrated higher-grade retrolisthesis. Accordingly, the present analysis focused on retrolisthesis burden, defined by the number and location of involved lumbar levels, rather than comparison across retrolisthesis severity grades.

### 2.7. Statistical Analysis

Patients were stratified into two groups based on whether they underwent revision surgery: a Revision group (n = 14) and a No Revision group (n = 102). Continuous variables were assessed for normality using the Shapiro–Wilk test. Normally distributed variables were compared using the independent-samples *t*-test. Non-normally distributed variables were compared using the Mann–Whitney U test. All continuous variables were reported as mean ± standard deviation (SD). Categorical variables were compared using Fisher’s exact test for 2 × 2 tables or when expected cell counts fell below 5 in larger tables.

Because follow-up time varied across patients and revision surgery is a delayed, time-dependent event, survival analysis was used as the primary analytic approach for evaluating predictors of revision. For patients who underwent revision, survival time was defined as the interval from index surgery to revision. For patients who did not undergo revision, survival time was censored at the most recent clinical encounter. This approach was selected to account for differential follow-up duration and to avoid treating patients with limited follow-up as equivalent to patients with long-term revision-free survival. The survival analysis included all 14 revision events in the analytic cohort of 116 patients. For patients with degenerative revisions, the survival time was defined as the interval from index surgery to revision. For all other patients, survival time was censored at the most recent clinical encounter. Hazard ratios (HR) with 95% confidence intervals (CI) were estimated using Cox proportional hazards regression. Standard errors were derived from the observed information matrix. Univariate Cox models were fit for all candidate predictors. Multivariate models were limited to two predictors given 14 events. Kaplan–Meier curves were used to visualize revision-free survival, and groups were compared using the log-rank test. Because no validated literature-based threshold exists for defining high-burden lumbar retrolisthesis in patients undergoing L4–L5 fusion, retrolisthesis burden was analyzed both continuously and categorically. The ≥3-level threshold was selected as an exploratory, distribution-based marker of high retrolisthesis burden, identifying patients with more diffuse multilevel posterior translation rather than absent, isolated, or limited retrolisthesis. Given the limited number of revision events, formal ROC-derived threshold selection was not performed, as this would risk overfitting and imply a level of cutoff precision not supported by the sample size. All tests were two-sided with significance set at *p* < 0.05. Analyses were performed using Python 3.12 with SciPy 1.14 (SciPy Foundation).

## 3. Results

### 3.1. Population Characteristics

The analytic cohort comprised 116 patients who underwent single-level L4–L5 posterior spinal fusion with multilevel decompressive laminectomy. The mean age was 63.5 ± 9.3 years (*n* = 83 with age recorded), and the cohort was predominantly White (84 patients, 72.4%) with 25 Black patients (21.6%). Women comprised 58.6% of the cohort. Mean BMI was 31.5 ± 6.3 kg/m^2^, and the mean Charlson Comorbidity Index was 1.2 ± 1.4. Diabetes mellitus was present in 20 patients (17.2%), hypertension in 72 (62.1%), and 8 patients (6.9%) had a documented history of osteoporosis. Baseline demographic and clinical characteristics are summarized in Table 1. There were no significant differences between the Revision and No Revision groups in age, sex, race, BMI, CCI, ASA class, smoking status, or individual comorbidities (all *p* > 0.05).

### 3.2. Surgical and Perioperative Characteristics

Operative time averaged 157.5 ± 48.1 min, and estimated blood loss averaged 295.1 ± 277.2 mL. Mean hospital length of stay was 4.0 ± 2.6 days. Intraoperative complications were recorded in 20 patients (17.2%), and in-hospital postoperative complications were recorded in 31 patients (26.7%). Surgical and perioperative characteristics did not differ significantly between the Revision and No Revision groups, including operative time, estimated blood loss, hospital length of stay, intraoperative complications, and hospital complications (all *p* > 0.05; Table 2).

### 3.3. Retrolisthesis Prevalence and Distribution

**Figure 1:**
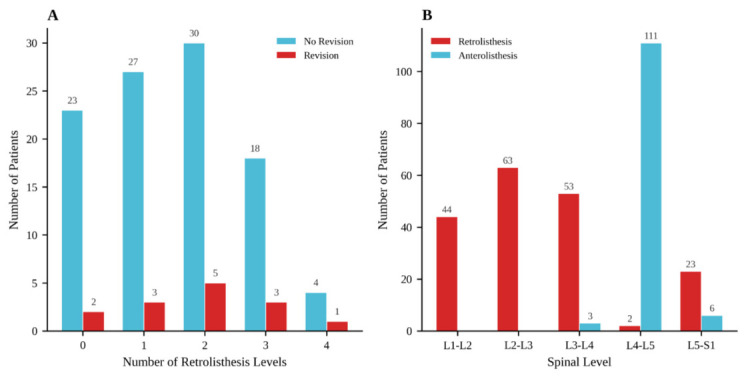
**Retrolisthesis Distribution in the Cohort.** (**A**) Distribution of the number of retrolisthesis levels per patient stratified by revision status. (**B**) Frequency of retrolisthesis and anterolisthesis at each lumbar motion segment across the full cohort.

Preoperative retrolisthesis was present in 91 of 116 patients (78.4%). Among patients with retrolisthesis, 30 (33.0%) had a single level involved, 35 (38.5%) had two levels, 21 (23.1%) had three levels, and 5 (5.5%) had four levels. The most commonly affected segments were L3–L4 (47.4% of patients) and L2–L3 (46.6%), followed by L1–L2 (34.5%) and L5–S1 (13.8%). Retrolisthesis at the L4–L5 level itself was observed in 4 patients (3.4%). Figure 1 displays the distribution of retrolisthetic levels across the cohort by revision status (Panel A) and by spinal level (Panel B).

There was no significant association between the presence of retrolisthesis and revision surgery. Retrolisthesis was present in 12 of 14 revision patients (85.7%) compared with 79 of 102 non-revision patients (77.5%, *p* = 0.73, Fisher’s exact test). The mean number of retrolisthetic levels was 1.8 ± 1.2 in the revision group versus 1.5 ± 1.2 in the no revision group (*p* = 0.15, Mann–Whitney U test). Adjacent retrolisthesis (L3–L4 or L5–S1) was present in 10 of 14 revision patients (71.4%) versus 55 of 102 non-revision patients (53.9%, *p* = 0.26). Retrolisthesis at L2–L3 was present in 8 of 14 revision patients (57.1%) versus 46 of 102 non-revision patients (45.1%, *p* = 0.41). Neither adjacent nor remote retrolisthesis was significantly associated with revision on Fisher’s exact testing (Table 3).

### 3.4. Spinopelvic Alignment

**Figure 2:**
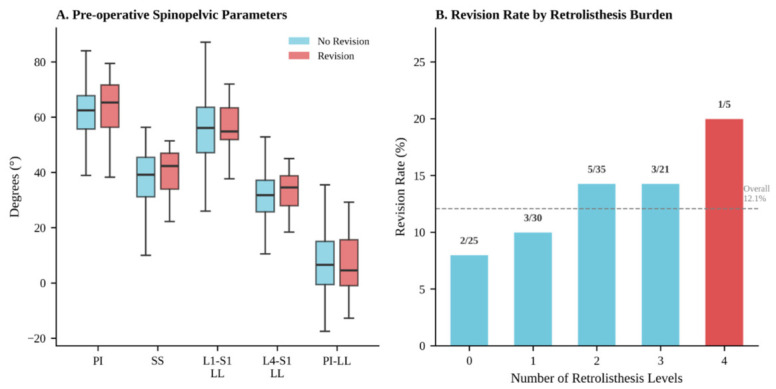
**Spinopelvic Parameters and Revision by Retrolisthesis Burden.** (**A**) Preoperative spinopelvic parameters (PI, SS, L1–S1 LL, L4–S1 LL, PI–LL mismatch) by revision status, shown as box plots. (**B**) Revision rate by number of retrolisthesis levels. Dashed line = overall cohort revision rate (12.1%).

Preoperative mean pelvic incidence was 55.8° ± 11.3°, sacral slope 34.6° ± 9.2°, L1–S1 lumbar lordosis 44.7° ± 12.4°, and L4-S1 segmental lordosis 28.4° ± 8.2°. The mean PI-LL mismatch was 11.0° ± 11.5°. No preoperative spinopelvic parameter differed significantly between the Revision and No Revision groups (all *p* > 0.05, Table 4). Figure 2A illustrates preoperative spinopelvic parameter distributions by revision status.

Immediate postoperative radiographic data were available for 75 patients (14 revision, 61 non-revision). The revision group demonstrated significantly greater postoperative L4–S1 lordosis (31.9° ± 5.8° vs. 26.5° ± 7.0°, *p* = 0.013 by Mann–Whitney U test), but did not differ on postoperative pelvic incidence, sacral slope, L1-S1 lordosis, or PI-LL mismatch. Change scores from preoperative to immediate postoperative measurements did not differ significantly between groups for any parameter. Specifically, PI-LL mismatch was not significantly different between the Revision and No Revision groups preoperatively, postoperatively, or when evaluated as a change score, suggesting that conventional global sagittal mismatch did not distinguish patients who later underwent revision in this cohort. Figure 2B displays revision rates stratified by the number of retrolisthetic levels.

### 3.5. Survival Analysis

Median follow-up for the cohort was 12.0 months (range 0.1 to 76.0). Follow-up was significantly shorter in the No Revision group than in the Revision group, reflecting the delayed nature of symptomatic adjacent segment disease requiring revision. Because unequal follow-up can bias cross-sectional comparisons by classifying patients with limited observation time as non-events, Kaplan–Meier analysis and Cox proportional hazards regression were used to evaluate revision-free survival with censoring at the most recent clinical encounter. Accordingly, the survival analyses should be interpreted as time-to-revision analyses rather than simple comparisons of revision proportions. All 14 revision events were performed for symptomatic adjacent segment disease. Each revision patient presented with radicular pain and underwent revision after clinical and radiographic evaluation demonstrated pathology warranting operative management.

**Figure 3:**
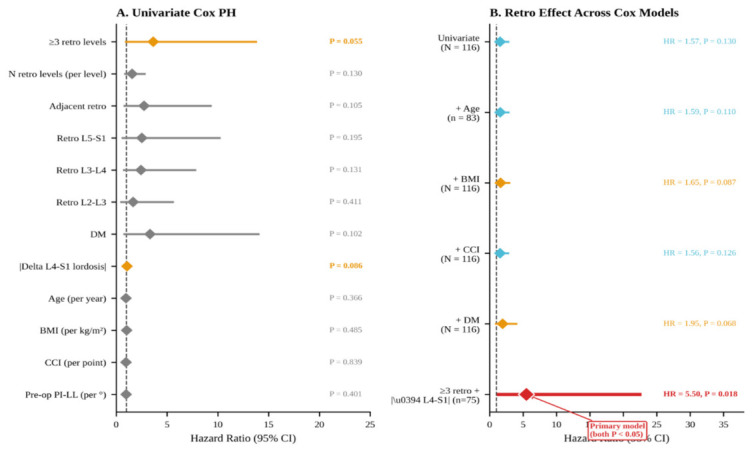
**Cox Proportional Hazards Analysis: Univariate and Multivariate Models.** (**A**) Univariate Cox proportional hazards analysis for key predictors of revision surgery. Diamonds indicate hazard ratios with 95% confidence intervals. Red = *p* < 0.05, orange = *p* < 0.10, gray = *p* ≥ 0.10. Dashed line = HR 1.0 (null). (**B**) Hazard ratio for retrolisthesis across multivariate Cox models. Models 1–4 show the N retro levels HR (continuous) adjusting for one covariate each. Model 5 (primary model, bottom) shows the ≥3 retro binary HR when combined with |Δ L4–S1 lordosis|. In Model 5, both predictors are independently significant: ≥3 retro levels (HR = 5.50, *p* = 0.018) and |Δ L4–S1 lordosis| (HR = 1.09 per degree, *p* = 0.031).

On univariate Cox proportional hazards analysis (14 revision events), the exploratory high-burden subgroup, defined as patients with three or more retrolisthesis levels, demonstrated an elevated hazard of revision (HR = 3.65, 95% CI 0.97 to 13.75, *p* = 0.055, log-rank *p* = 0.040). A dose–response trend was observed with the number of retrolisthesis levels as a continuous predictor, though this did not reach significance (HR = 1.57 per additional level, 95% CI 0.88 to 2.80, *p* = 0.15). Adjacent retrolisthesis (L3–L4 or L5–S1) trended toward significance (HR = 2.47, 95% CI 0.71 to 8.61, *p* = 0.16), as did retrolisthesis at L5–S1 (HR = 3.02, *p* = 0.13) and L3-L4 (HR = 2.14, *p* = 0.21). Diabetes mellitus trended toward significance on univariate Cox analysis (HR = 3.32, 95% CI 0.79 to 13.99, *p* = 0.10). No preoperative radiographic parameter or perioperative variable was significantly associated with revision. Full univariate Cox results are presented in Table 5 and Figure 3A. Consistent with the groupwise comparisons, preoperative PI-LL mismatch was not associated with revision on Cox regression, whereas the strongest radiographic signal emerged from the absolute magnitude of L4-S1 segmental lordosis change in the postoperative radiographic subset.

**Figure 4:**
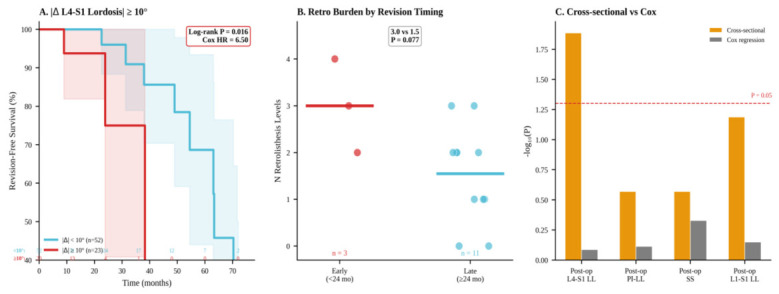
**L4–S1 Lordosis Correction, Revision Timing, and Cross-Sectional vs. Survival Analysis.** (**A**) Kaplan–Meier revision-free survival by absolute magnitude of L4–S1 lordosis correction. Patients with |Δ L4–S1 lordosis| ≥ 10° (red) had significantly lower revision-free survival (log-rank *p* = 0.016, Cox HR = 6.50). (**B**) Number of retrolisthesis levels in early (<24 months, *n* = 3) vs. late (≥24 months, *n* = 11) revision patients. Horizontal lines indicate group means. Early revisions had higher retrolisthesis burden (3.0 vs. 1.5, *p* = 0.077). (**C**) Comparison of *p* values from cross-sectional analysis (Mann–Whitney U) vs. Cox regression for postoperative radiographic parameters. Post-operative L4–S1 lordosis appeared significant cross-sectionally (*p* = 0.013) but was null on Cox regression (*p* = 0.82), demonstrating the impact of differential follow-up and selection bias. Red dashed line = *p* = 0.05 threshold.

Among 75 patients with postoperative L4–S1 lordosis measurements, a two-predictor Cox model combining three or more retrolisthesis levels with the absolute magnitude of L4–S1 lordosis correction yielded two independently significant predictors of revision. Patients with three or more retrolisthesis levels had a 5.5-fold increased hazard (HR = 5.50, 95% CI 1.35 to 22.46, *p* = 0.018), and each additional degree of absolute L4–S1 lordosis change was associated with a 9% increase in the hazard of revision (HR = 1.09, 95% CI 1.01 to 1.18, *p* = 0.031). This was the only model in which both predictors reached significance. Absolute lordosis change captures the magnitude of segmental correction at the fusion level regardless of direction (loss or gain), reflecting surgical alteration of the local biomechanical environment. When dichotomized at 10 degrees, patients with |Δ L4-S1 lordosis| ≥ 10° had significantly lower revision-free survival (log-rank *p* = 0.016, Cox HR = 6.50, Figure 4A).

Additional multivariate models demonstrated a consistent effect of retrolisthesis burden across covariates. The number of retrolisthesis levels showed persistent trends when adjusting for age (HR = 1.59, *p* = 0.11), BMI (HR = 1.65, *p* = 0.087), or CCI (HR = 1.56, *p* = 0.14). When adjusting for diabetes mellitus, the retro effect strengthened (HR = 1.95, *p* = 0.068), and diabetes was independently associated with revision (HR = 5.55, 95% CI 1.09 to 28.23, *p* = 0.039). Table 6 and Figure 3B present all multivariate models.

Cross-sectional comparison had suggested that postoperative L4–S1 lordosis differed between groups (31.9° vs. 26.5°, *p* = 0.013 by Mann–Whitney U). However, when analyzed with Cox regression accounting for censoring, postoperative L4–S1 lordosis was not a significant predictor of revision (HR = 0.99, *p* = 0.82), nor was any other postoperative radiographic parameter (all *p* > 0.40). This discrepancy reflects differential follow-up: postoperative imaging was available for all 14 revision patients but only 61 of 102 non-revision patients, creating selection bias in the cross-sectional comparison (Figure 4C).

**Figure 5:**
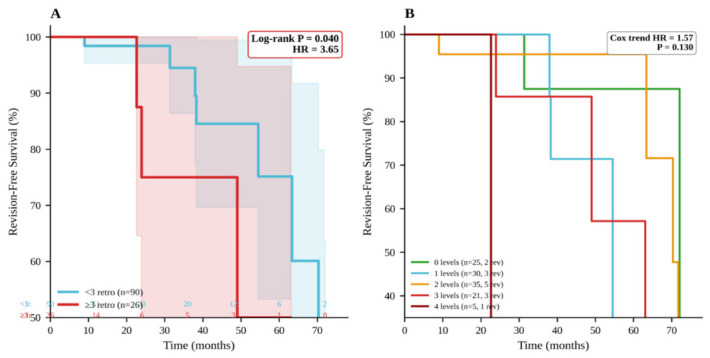
**Kaplan–Meier Revision-Free Survival by Retrolisthesis Burden.** Kaplan–Meier revision-free survival curves. (**A**) Patients with ≥3 retrolisthesis levels (red) versus <3 levels (teal). Shaded regions = 95% confidence intervals. Numbers at risk shown below. Patients with ≥3 levels had a 3.7-fold increased hazard (HR = 3.65, 95% CI 0.97–13.75, Cox *p* = 0.055, log-rank *p* = 0.040). (**B**) Survival stratified by number of retrolisthesis levels (0 through 4). A dose–response pattern was observed (HR = 1.57 per level, *p* = 0.15).

Kaplan–Meier revision-free survival curves stratified by retrolisthesis burden are shown in Figure 5. Patients with three or more retrolisthesis levels demonstrated markedly lower revision-free survival compared with those with fewer than three levels (Panel A, log-rank *p* = 0.040). A dose–response pattern was observed across individual-level groups (0 through 4 retrolisthesis levels, Panel B), with progressive separation of curves beyond 24 months.

Among the 14 revision patients, 3 underwent early revision (less than 24 months from index surgery) and 11 underwent late revision (24 months or later). Early revision patients had a higher mean number of retrolisthesis levels (3.0 ± 0.8 vs. 1.5 ± 1.0, *p* = 0.077), suggesting that greater retrolisthesis burden may accelerate time to failure (Figure 4B). This pattern is consistent with the observation that adjacent segment disease, the predominant revision indication, is a late-onset complication whose development may be hastened by pre-existing multilevel segmental instability. All revision patients underwent surgery for symptomatic adjacent segment disease/adjacent stenosis, and all presented with radicular pain prior to revision. (Table 7) Mean time to revision was 48.4 ± 21.4 months, with 3 patients undergoing early revision within 24 months and 11 patients undergoing late revision at 24 months or later. On paired radiographic comparison between immediate postoperative and pre-revision imaging, L4–S1 lordosis decreased significantly from 31.9° ± 6.0° to 25.6° ± 8.3° (*p* = 0.01), while sacral slope and PI-LL mismatch did not demonstrate statistically significant changes. These findings further support that revision events represented clinically symptomatic adjacent segment pathology with corresponding radiographic evaluation rather than nonspecific reoperations.

**Figure 6:**
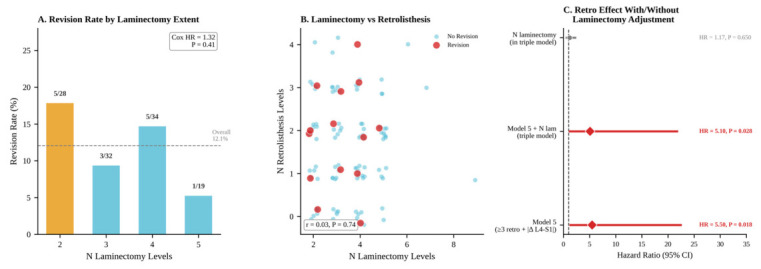
**Sensitivity Analysis: Laminectomy Extent as a Potential Confounder.** Sensitivity Analysis: Laminectomy Extent as a Potential Confounder. (**A**) Revision rate by number of laminectomy levels. No significant trend was observed (Cox HR = 1.32, 95% CI 0.68–2.54, *p* = 0.41). The dashed line indicates the overall cohort revision rate (12.1%). (**B**) Scatter plot of the number of laminectomy levels versus the number of retrolisthesis levels for each patient. Red dots indicate patients who underwent revision surgery. No correlation was observed between laminectomy extent and retrolisthesis burden (Spearman r = 0.03, *p* = 0.74). (**C**) Forest plot comparing the primary multivariate model (Model 5: ≥3 retrolisthesis levels + |Δ L4–S1 lordosis|) with and without adjustment for laminectomy extent. Both retrolisthesis burden (HR = 5.10, *p* = 0.028) and absolute L4–S1 lordosis change (HR = 1.09, *p* = 0.033) remained significant after adding laminectomy extent, while laminectomy itself was not a significant predictor (HR = 1.17, *p* = 0.65).

To evaluate whether decompression extent confounded the relationship between retrolisthesis burden and revision, we performed an additional sensitivity analysis using the number of laminectomy levels recorded from operative reports. The number of laminectomy levels was not significantly associated with revision surgery on Cox proportional hazards analysis (HR = 1.32, 95% CI 0.68–2.54, *p* = 0.41). Revision rates varied across laminectomy extent groups, but no significant trend was observed. In addition, laminectomy extent was not correlated with retrolisthesis burden (Spearman r = 0.03, *p* = 0.74), suggesting that patients with greater retrolisthesis burden did not simply undergo more extensive decompression. When laminectomy extent was added to the primary multivariate model containing ≥3 retrolisthesis levels and absolute L4–S1 lordosis change, both ≥3 retrolisthesis levels (HR = 5.10, *p* = 0.028) and absolute L4–S1 lordosis change (HR = 1.09 per degree, *p* = 0.033) remained significant, while laminectomy extent was not independently associated with revision (HR = 1.17, *p* = 0.65; Figure 6).

## 4. Discussion

In this cohort, preoperative lumbar retrolisthesis was highly prevalent among patients undergoing single-level L4–L5 fusion for degenerative pathology, occurring in 91 of 116 patients (78.4%). Notably, this was usually not a single-level finding: among patients with retrolisthesis, 67.0% had involvement at 2 or more lumbar levels, with the greatest burden at L3–L4 (47.4%) and L2–L3 (46.6%), whereas retrolisthesis at the index L4–L5 level itself was uncommon (3.4%). This pattern is broadly consistent with the existing lumbar spine literature, which has generally suggested that retrolisthesis is less common than anterolisthesis in broader radiographic populations, tends to occur more often at cephalad lumbar levels such as L3–L4 rather than predominantly at L4–L5, and is frequently encountered in the setting of multilevel degenerative change rather than as an isolated index-level abnormality [17,19,20]. Prior radiographic studies have further interpreted lumbar retrolisthesis as a compensatory or degenerative phenomenon associated with altered sagittal alignment and relatively low pelvic incidence, rather than a purely focal translational event [15,16]. Within that context, the particularly high prevalence and multilevel distribution observed in our study likely reflect the surgically selected nature of this L4–L5 fusion population, in whom advanced multilevel degeneration and compensatory biomechanical remodeling would be expected to be enriched. Taken together, our findings suggest that in patients undergoing L4–L5 fusion, retrolisthesis may be better understood as a marker of a more globally degenerative and mechanically vulnerable lumbar spine than as an incidental radiographic finding confined to the operative segment.

Preoperative retrolisthesis was not associated with revision when assessed as a simple binary variable, as any retrolisthesis was common in both revision and non-revision patients (85.7% vs. 77.5%; *p* = 0.73), and neither adjacent nor remote retrolisthesis differed significantly on groupwise comparison. However, when revision was analyzed as a time-dependent outcome, the retrolisthesis burden appeared to be more informative than retrolisthesis presence alone. Patients with 3 or more retrolisthesis levels demonstrated inferior revision-free survival, with a 3.65-fold higher hazard of revision on univariate Cox analysis, and in the postoperative radiographic subset, 3 or more retrolisthesis levels and greater absolute L4–S1 lordosis change were each independently associated with revision. In that model, 3 or more retrolisthesis levels were associated with a 5.50-fold increased hazard of revision, while each additional degree of absolute L4–S1 lordosis change increased revision hazard by 9%, suggesting that greater local correction may amplify failure risk in a spine already marked by substantial multilevel retrolisthesis burden. Importantly, the ≥3-level threshold should be interpreted as an exploratory, distribution-based marker of high retrolisthesis burden rather than as a validated clinical cutoff. Prior literature supports retrolisthesis as a degenerative and compensatory radiographic phenomenon that may occur across multiple lumbar levels, but no established number-of-level threshold has been validated for predicting revision after single-level L4–L5 fusion [16,19,20]. Therefore, this finding should be interpreted as hypothesis-generating and should be validated in larger cohorts with longer follow-up. This distinction is clinically relevant and aligns with the broader lumbar fusion literature, which has consistently shown that adjacent segment failure is multifactorial and often delayed, rather than attributable to a single radiographic feature in isolation. Prior studies have more reliably implicated the combined effects of baseline degeneration, altered adjacent-level mechanics, and sagittal alignment parameters such as PI-LL mismatch and lordosis restoration in the development of symptomatic ASD and revision after lumbar fusion [10,11,13,21,22]. Within that framework, our findings suggest that retrolisthesis may be too prevalent in a degenerative L4–L5 fusion population to function as a discriminating yes-or-no marker, whereas increasing multilevel retrolisthesis burden may better identify a spine with more diffuse pre-existing degeneration and diminished biomechanical reserve. This interpretation is further supported by the limited non-fusion literature, as Ikegami et al. found that preoperative retrolisthesis predicted delayed reoperation after lumbar decompression for foraminal stenosis, suggesting that posterior translational change may mark occult segmental vulnerability even before fusion is introduced [23].

Global sagittal alignment, particularly PI-LL mismatch, has been repeatedly implicated in the development of adjacent segment disease after lumbar fusion. Prior studies have demonstrated that elevated PI-LL mismatch is associated with symptomatic adjacent-level disease, adjacent segment degeneration, and revision after short-segment lumbar fusion [12,13,14]. Accordingly, our findings should not be interpreted as diminishing the importance of global sagittal alignment. Rather, in the present cohort of patients undergoing single-level L4–L5 fusion, conventional global spinopelvic measures, including pelvic incidence, sacral slope, L1–S1 lumbar lordosis, and PI-LL mismatch, did not significantly differ between revision and non-revision patients and were not independently associated with revision. In contrast, the strongest radiographic signal was observed for the absolute magnitude of L4–S1 segmental lordosis change, particularly when considered alongside multilevel retrolisthesis burden. This distinction may be clinically relevant. PI-LL mismatch captures global harmony between pelvic morphology and lumbar lordosis, whereas L4–S1 lordosis change reflects the magnitude of local correction imposed across the distal lumbar spine. Because the present cohort was limited to single-level L4–L5 fusion, revision risk may have been more sensitive to local changes in the distal lumbar mechanical environment than to global sagittal mismatch alone. Prior work has similarly emphasized that postoperative durability depends not only on total lumbar lordosis, but also on how lordosis is restored and distributed across the lower lumbar spine, with segmental lordosis, distal lumbar lordosis, and L4–S1 lordosis restoration all linked to adjacent segment pathology and reoperation risk [24,25,26]. Within this framework, multilevel retrolisthesis may represent a visible radiographic marker of a spine that is already compensating across multiple segments, making it less tolerant of larger local correction at L4–S1 even in the absence of a significant baseline PI-LL mismatch.

This interpretation is supported by prior lumbar spine literature, which has characterized retrolisthesis as a compensatory mechanism associated with low pelvic incidence, sagittal imbalance, and posterior translational remodeling in degenerative spines [15,16,27,28]. In parallel, the lumbar fusion literature increasingly suggests that postoperative durability depends not only on global alignment, but also on how lordosis is restored and distributed across the distal lumbar spine, with ideal lordosis correction, change in segmental lordosis, distal lumbar lordosis, and L4–S1 lordosis restoration all linked to adjacent segment pathology and reoperation risk [24,25,26,29]. Within that framework, our findings suggest that the clinically relevant issue may not be whether standard global radiographic parameters differ at baseline, but whether a spine already demonstrating multilevel compensatory retrolisthesis can tolerate the magnitude of local correction imposed at L4–S1. This may explain why retrolisthesis burden alone carried a revision signal on survival analysis and why that signal strengthened further when greater absolute L4-S1 lordosis change was considered concurrently, with patients with 3 or more retrolisthesis levels demonstrating a 5.50-fold increased hazard of revision and each additional degree of absolute L4–S1 lordosis change increasing revision hazard by 9%. In that sense, multilevel retrolisthesis may represent a visible radiographic signature of a spine that is already compensating across multiple segments and is therefore less tolerant of larger distal segmental correction, predisposing to later symptomatic revision for adjacent segment disease. The extent of decompression is also important when interpreting revision risk after lumbar fusion, as multilevel laminectomy may alter posterior element integrity and contribute to postoperative segmental instability. In response to this concern, we performed a sensitivity analysis evaluating the number of laminectomy levels as a potential confounder. Laminectomy extent was not significantly associated with revision, was not correlated with retrolisthesis burden, and did not eliminate the observed associations between ≥3 retrolisthesis levels, absolute L4-S1 lordosis change, and revision. These findings suggest that the revision signal observed in patients with greater retrolisthesis burden was not solely attributable to undergoing more extensive decompression. However, this analysis should be interpreted as exploratory, as the number of laminectomy levels may not fully capture decompression morphology, including facet resection, ligamentous disruption, or unilateral versus bilateral decompression.

This study has several limitations. First, it is a retrospective single-center analysis of patients undergoing single-level L4–L5 posterior fusion with multilevel decompression for degenerative pathology, which introduces the potential for selection bias and may limit generalizability to other lumbar fusion populations. Second, although all patients had postoperative clinical follow-up and revision was assessed as a symptomatic, clinically meaningful endpoint, follow-up duration was not uniform across the cohort, and later failures may not have been captured in all patients despite the use of survival analysis to account for censoring, survival analysis cannot fully eliminate the possibility that patients with shorter follow-up may later develop symptomatic adjacent segment disease requiring revision. Therefore, the present findings should be interpreted as associations with observed revision-free survival rather than definitive lifetime revision risk. Third, although all patients had postoperative clinical follow-up and revision was assessed as a symptomatic, clinically meaningful endpoint, follow-up duration was not uniform across the cohort, and later failures may not have been captured in all patients despite the use of survival analysis to account for censoring. Importantly, all revision events in this cohort were performed for symptomatic adjacent segment disease in patients presenting with radicular pain; however, a revision-based endpoint does not capture the full clinical spectrum of adjacent segment pathology, including patients with symptomatic disease managed nonoperatively. In addition, patient-reported outcome measures, including VAS pain scores and ODI, were not consistently available, and granular motor examination data, such as standardized documentation of motor strength deficits, were not uniformly recorded. Future studies incorporating longitudinal pain, disability, functional, and neurologic outcomes are needed to determine whether multilevel retrolisthesis burden is also associated with symptomatic deterioration that does not progress to reoperation. Immediate postoperative radiographic data were also available for only a subset of patients, which may have influenced postoperative alignment comparisons. Moreover, the laminectomy extent was evaluated in sensitivity analysis; it was measured using the number of decompressed levels and may not fully capture decompression morphology, including the degree of facet resection, preservation of posterior ligamentous structures, or unilateral versus bilateral decompression. Therefore, while the laminectomy extent did not appear to explain the observed retrolisthesis signal, future studies should evaluate decompression characteristics in greater detail. Retrolisthesis and sagittal alignment parameters were assessed on standing lateral radiographs, which capture static posterior translation and alignment but do not fully characterize dynamic instability. Because flexion–extension radiographs were not uniformly available, we could not systematically evaluate segmental mobility at the L4–L5 degenerative spondylolisthesis level or at adjacent retrolisthesis levels. In addition, all retrolisthesis identified in this cohort were Grade I, which limited the ability to evaluate whether increasing retrolisthesis severity is associated with revision risk. Therefore, the present findings reflect the prognostic relevance of multilevel low-grade retrolisthesis burden and distribution rather than the independent effect of higher-grade posterior translation at an individual motion segment. Moreover, the ≥3-level retrolisthesis threshold should also be interpreted as exploratory and distribution-based rather than as a validated clinical cutoff. Although retrolisthesis burden was evaluated continuously as well as categorically, the small number of revision events limited formal threshold derivation, and larger cohorts are needed to determine whether a reproducible retrolisthesis burden threshold exists and whether this threshold remains associated with revision risk after adjustment for additional clinical and radiographic factors. Finally, although PI-LL mismatch and other global spinopelvic parameters were evaluated, this study was not powered to perform extensive multivariable modeling of multiple interrelated sagittal alignment variables. Therefore, the absence of a significant association between PI-LL mismatch and revision in this cohort should be interpreted cautiously and should not be viewed as contradicting prior literature establishing PI-LL mismatch as an important risk factor for adjacent segment disease. Larger studies with full-length standing radiographs, longer follow-up, and more revision events are needed to determine whether retrolisthesis burden modifies the relationship between global sagittal alignment, distal lordosis correction, and adjacent segment failure. Nonetheless, the cohort was radiographically well characterized, preoperative measurements were available for all patients, and revision was evaluated with time-to-event methods appropriate for a delayed outcome such as symptomatic adjacent segment failure.

## 5. Conclusions

In patients undergoing single-level L4–L5 fusion for degenerative pathology, preoperative retrolisthesis alone did not differentiate revision risk; however, a greater burden of lumbar retrolisthesis was associated with worse revision-free survival, particularly when accompanied by greater change in L4–S1 lordosis. These findings suggest that in patients undergoing single-level L4–L5 fusion, often for degenerative spondylolisthesis, multilevel retrolisthesis with greater local sagittal correction may represent a mechanically vulnerable spine phenotype associated with later symptomatic revision for adjacent segment disease. Given the modest sample size and limited number of revision events, these findings should be considered hypothesis-generating and require validation in larger cohorts with longer follow-up. Because the present analysis focused on symptomatic ASD requiring revision surgery, future studies with longitudinal PROs and standardized neurologic assessments are needed to better define the relationship between the retrolisthesis burden and nonoperative symptomatic adjacent segment pathology.

## Figures and Tables

**Table 1:** Baseline Demographic and Clinical Characteristics.

Variable	Overall (*n* = 116)	Revision (*n* = 14)	No Revision (*n* = 102)	*p* Value
**Age (years)**	**63.5 ± 9.3**	**57.8 ± 8.9**	**64.7 ± 9.0**	**0.01**
BMI (kg/m^2^)	30.3 ± 6.3	31.3 ± 5.5	30.1 ± 6.4	0.37
CCI	2.7 ± 1.8	2.2 ± 1.8	2.8 ± 1.8	0.33
**Follow-up (months)**	**20.3 ± 18.7**	**44.2 ± 20.9**	**17.0 ± 15.9**	**<0.001**
***Sex***				
Female	76 (65.5)	11 (78.6)	65 (63.7)	
Male	40 (34.5)	3 (21.4)	37 (36.3)	
***Race***				
White	83 (71.6)	11 (78.6)	72 (70.6)	
Black	29 (25.0)	3 (21.4)	26 (25.5)	
Hispanic	2 (1.7)	0 (0.0)	2 (2.0)	
Asian	1 (0.9)	0 (0.0)	1 (1.0)	
Native American	1 (0.9)	0 (0.0)	1 (1.0)	
***ASA Class***				
2	31 (26.7)	6 (42.9)	25 (24.5)	
3	83 (71.6)	8 (57.1)	75 (73.5)	
4	2 (1.7)	0 (0.0)	2 (2.0)	
***Symptoms***				
Back pain	110 (94.8)	14 (100.0)	96 (94.1)	1.00
Leg pain	107 (92.2)	13 (92.9)	94 (92.2)	1.00
Bilateral leg symptoms	70 (60.3)	9 (64.3)	61 (59.8)	1.00
Weakness	40 (34.5)	2 (14.3)	38 (37.3)	0.13
***Smoking Status***				
Current smoker	13 (11.2)	0 (0.0)	13 (12.7)	0.36
Former smoker	47 (40.5)	6 (42.9)	41 (40.2)	1.00
***Comorbidities***				
Diabetes mellitus	20 (17.2)	3 (21.4)	17 (16.7)	0.71
Hypertension	81 (69.8)	9 (64.3)	72 (70.6)	0.76
Osteoporosis	9 (7.8)	1 (7.1)	8 (7.8)	1.00
History of cancer	22 (19.0)	2 (14.3)	20 (19.6)	1.00
Chronic steroid use	16 (13.8)	1 (7.1)	15 (14.7)	0.69
Kidney disease	8 (6.9)	1 (7.1)	7 (6.9)	1.00
Previous spine surgery	25 (21.6)	3 (21.4)	22 (21.6)	1.00

Continuous variables are presented as mean ± standard deviation. Categorical variables are presented as *n* (%). Bold indicates *p* < 0.05. CCI, Charlson Comorbidity Index. ASA, American Society of Anesthesiologists. Age available for 83 of 116 patients (71.6%).

**Table 2:** Surgical and Perioperative Characteristics.

Variable	Overall (*n* = 116)	Revision (*n* = 14)	No Revision (*n* = 102)	*p* Value
Operative time (min)	157.5 ± 48.1	150.1 ± 42.8	158.5 ± 48.9	0.44
EBL (mL)	295.1 ± 277.2	296.4 ± 192.6	295.0 ± 287.6	0.48
Hospital LOS (days)	4.0 ± 2.6	3.4 ± 1.3	4.1 ± 2.7	0.64
Intraoperative complications	20 (17.2)	2 (14.3)	18 (17.6)	1.00
Hospital complications	31 (26.7)	4 (28.6)	27 (26.5)	1.00

EBL, estimated blood loss. LOS, length of stay.

**Table 3:** Retrolisthesis and Anterolisthesis Distribution.

Variable	Overall (*n* = 116)	Revision (*n* = 14)	No Revision (*n* = 102)	*p* Value
***Number of retrolisthesis levels***				
0	25 (21.6)	2 (14.3)	23 (22.5)	
1	30 (25.9)	3 (21.4)	27 (26.5)	
2	35 (30.2)	5 (35.7)	30 (29.4)	
3	21 (18.1)	3 (21.4)	18 (17.6)	
4	5 (4.3)	1 (7.1)	4 (3.9)	
***Any retrolisthesis/location***				
Any retrolisthesis	91 (78.4)	12 (85.7)	79 (77.5)	0.73
Retrolisthesis at L1–L2	44 (37.9)	6 (42.9)	38 (37.3)	0.77
Retrolisthesis at L2–L3	63 (54.3)	10 (71.4)	53 (52.0)	0.25
Retrolisthesis at L3–L4	53 (45.7)	7 (50.0)	46 (45.1)	0.78
Retrolisthesis at L4–L5	2 (1.7)	0 (0.0)	2 (2.0)	1.00
Retrolisthesis at L5–S1	23 (19.8)	3 (21.4)	20 (19.6)	1.00
***Composite***				
Adjacent retro (L3–L4 or L5–S1)	65 (56.0)	8 (57.1)	57 (55.9)	1.00
Remote retro (L1–L2 or L2–L3)	72 (62.1)	11 (78.6)	61 (59.8)	0.24
***Anterolisthesis***				
Anterolisthesis at L3–L4	3 (2.6)	0 (0.0)	3 (2.9)	1.00
Anterolisthesis at L4–L5	111 (95.7)	14 (100.0)	97 (95.1)	1.00
Anterolisthesis at L5–S1	6 (5.2)	2 (14.3)	4 (3.9)	0.15

Adjacent = L3–L4 or L5–S1 (adjacent to the L4–L5 fusion). Remote = L1–L2 or L2–L3. Bold indicates *p* < 0.05.

**Table 4:** Spinopelvic Radiographic Parameters.

Variable	Overall (*n* = 116)	Revision (*n* = 14)	No Revision (*n* = 102)	*p* Value
***Preoperative***				
Pre-op Pelvic Incidence (°)	62.3 ± 11.1	62.6 ± 12.5	62.3 ± 10.9	0.94
Pre-op Sacral Slope (°)	38.9 ± 9.7	40.6 ± 8.5	38.7 ± 9.8	0.50
Pre-op L1–S1 Lordosis (°)	55.4 ± 12.3	56.0 ± 9.6	55.3 ± 12.6	0.83
Pre-op L4–S1 Lordosis (°)	31.7 ± 9.6	33.3 ± 8.1	31.5 ± 9.8	0.51
Pre-op PI-LL Mismatch (°)	7.0 ± 12.5	6.5 ± 13.1	7.0 ± 12.4	0.89
Postoperative (n = 75)				
Post-op Sacral Slope (°)	32.8 ± 10.0	35.1 ± 6.3	32.3 ± 10.6	0.60
Post-op L1–S1 Lordosis (°)	47.2 ± 10.3	50.9 ± 7.5	46.3 ± 10.7	0.13
**Post-op L4–S1 Lordosis (°)**	**27.5 ± 7.1**	**31.9 ± 6.0**	**26.5 ± 7.0**	**0.010**
Post-op PI-LL Mismatch (°)	16.2 ± 12.9	11.6 ± 14.2	17.3 ± 12.4	0.14
***Change (postop − preop, n = 75)***				
Δ Sacral Slope (°)	−7.1 ± 9.6	−5.4 ± 6.5	−7.5 ± 10.2	0.57
Δ L1–S1 Lordosis (°)	−9.2 ± 11.0	−5.1 ± 9.7	−10.1 ± 11.2	0.14
Δ L4–S1 Lordosis (°)	−4.5 ± 10.8	−1.5 ± 10.0	−5.2 ± 11.0	0.26
Δ PI-LL Mismatch (°)	9.2 ± 11.0	5.1 ± 9.7	10.1 ± 11.2	0.14

All values in degrees. PI, pelvic incidence. SS, sacral slope. LL, lumbar lordosis. Preoperative measurements available for all 116 patients, postoperative measurements available for 75. PI is an anatomical constant (post-op PI = pre-op PI), and PI and delta PI rows are omitted from the post-op section. Bold indicates *p* < 0.05.

**Table 5:** Univariate Cox Proportional Hazards Analysis for Revision Surgery.

Variable	*n*	HR	95% CI	*p* Value
***Retrolisthesis***				
N retro levels (per level)	116	1.57	0.88–2.80	0.13
≥3 retro levels	116	3.65	0.97–13.75	0.06
Any retrolisthesis	116	2.21	0.48–10.19	0.31
Adjacent retro (L3–L4 or L5–S1)	116	2.75	0.81–9.30	0.10
Remote retro (L1–L2 or L2–L3)	116	1.91	0.52–7.08	0.33
Retro at L2–L3	116	1.66	0.49–5.58	0.41
Retro at L3–L4	116	2.44	0.77–7.77	0.13
Retro at L5–S1	116	2.52	0.62–10.17	0.19
***Demographics/Comorbidities***				
Age (per year)	83	0.97	0.90–1.04	0.37
Male sex	116	0.95	0.25–3.53	0.94
BMI (per kg/m^2^)	116	1.03	0.94–1.13	0.48
CCI (per point)	116	0.96	0.67–1.38	0.84
ASA class	116	0.78	0.25–2.43	0.66
Diabetes mellitus	116	3.32	0.79–13.99	0.10
Hypertension	116	1.05	0.31–3.54	0.93
Osteoporosis	116	0.61	0.08–4.89	0.64
History of cancer	116	0.56	0.12–2.65	0.47
Previous spine surgery	114	2.00	0.48–8.39	0.34
Weakness	116	0.75	0.15–3.73	0.72
Former smoker	116	0.54	0.17–1.73	0.30
***Radiographic/Surgical***				
Pre-op PI (per °)	116	0.99	0.94–1.04	0.63
Pre-op SS (per °)	116	1.02	0.96–1.10	0.50
Pre-op L1–S1 LL (per °)	116	1.02	0.96–1.08	0.49
Pre-op L4–S1 LL (per °)	116	1.00	0.92–1.09	0.98
Pre-op PI-LL (per °)	116	0.98	0.95–1.02	0.40
|Δ L4–S1 lordosis| (per °)	75	1.07	0.99–1.15	0.09
Op time (per min)	115	1.00	0.98–1.01	0.75
EBL (per mL)	116	1.00	1.00–1.00	0.51
LOS (per day)	116	1.11	0.85–1.44	0.46

HR, hazard ratio. CI, confidence interval. Bold indicates *p* < 0.05. Cox regression accounts for differential follow-up via censoring.

**Table 6:** Multivariate Cox Proportional Hazards Models.

Variable	HR	95% CI	*p* Value
***Model 1: N retro + Age (n = 83)***			
N retro levels	1.59	0.90–2.82	0.11
Age (per year)	0.96	0.89–1.03	0.25
***Model 2: N retro + BMI (n = 116)***			
N retro levels	1.65	0.93–2.92	0.09
BMI (per kg/m^2^)	1.05	0.96–1.16	0.28
***Model 3: N retro + CCI (n = 116)***			
N retro levels	1.56	0.88–2.77	0.13
CCI (per point)	0.95	0.66–1.36	0.77
***Model 4: N retro + DM (n = 116)***			
N retro levels	1.95	0.95–3.98	0.07
** ** **Diabetes mellitus**	**5.55**	**1.09–28.23**	**0.04**
***Model 5: ≥3 retro + |Δ L4-S1 lordosis| (n = 75)***			
** ** **≥3 retro levels**	**5.50**	**1.35–22.46**	**0.02**
** ** **|Δ L4–S1 lordosis| (per °)**	**1.09**	**1.01–1.18**	**0.03**

Each model includes two predictors. Bold indicates *p* < 0.05. Model 1 reduced to *n* = 83 (missing age). Models 2–4 use *n* = 116. Model 5 reduced to *n* = 75 (patients with postoperative L4–S1 lordosis measurements). |Δ L4–S1 lordosis| = absolute change in L4–S1 segmental lordosis from pre- to postoperative measurement.

**Table 7:** Characterization of Revision Surgery Patients (n = 14).

Variable	*n* (%)	Mean ± SD	
***Presenting Symptoms at Index Surgery (n = 14)***			
Back pain	14 (100.0)		
Leg pain	13 (92.9)		
Motor weakness	2 (14.3)		
Bilateral leg symptoms	9 (64.3)		
***Revision Timing (n = 14)***			
Time to revision (months)		48.4 ± 21.4	
Early revision (<24 months)	3 (21.4)		
Late revision (≥24 months)	11 (78.6)		
***Radiographic Trajectory***	**Immed Post-op**	**Pre-Revision**	***p*** **Value**
Sacral slope (°)	34.9 ± 4.3	33.3 ± 5.5	0.17
**L4–S1 lordosis (°)**	**31.9 ± 6.0**	**25.6 ± 8.3**	**0.01**
PI-LL mismatch (°)	12.7 ± 12.0	18.4 ± 12.4	0.07

Categorical variables presented as *n* (%). Continuous variables presented as mean ± SD. Pre-revision radiographic measurements obtained at most recent imaging prior to revision surgery. Bold indicates *p* < 0.05 by the Wilcoxon signed-rank test (paired comparison). Revision patients lost a mean of 6.3° of L4–S1 segmental lordosis between immediate postoperative and pre-revision imaging.

## Data Availability

Data are available upon request from the authors.

## Abbreviations

The following abbreviations are used in this manuscript:

ASD	Adjacent Segment Disease
PI-LL Mismatch	Pelvic Incidence-Lumbar Lordosis Mismatch
EBL	Estimated Blood Loss
BMI	Body Mass Index
CCI	Charleson Comorbidity Index
ASA	American Society of Anesthesiologists
LOS	Length of Stay
HR	Hazard Ratio
CI	Confidence Interval
